# Effect of Ultrasonography-Guided Corticosteroid Injection vs Placebo Added to Exercise Therapy for Achilles Tendinopathy: A Randomized Clinical Trial

**DOI:** 10.1001/jamanetworkopen.2022.19661

**Published:** 2022-07-11

**Authors:** Finn Johannsen, Jens Lykkegaard Olesen, Tommy Frisgaard Øhlenschläger, Mathilde Lundgaard-Nielsen, Camilla Kjaer Cullum, Anna Svarre Jakobsen, Michael Skovdal Rathleff, Peter Stig Magnusson, Michael Kjær

**Affiliations:** 1 Institute of Sports Medicine, Department of Orthopedic Surgery M, Bispebjerg Hospital, Copenhagen, Denmark; 2 Center for General Practice at Aalborg University, Aalborg University, Aalborg, Denmark; 3 Department of Occupational Therapy and Physiotherapy, Bispebjerg Hospital, Copenhagen, Denmark; 4 Center for Rheumatology and Spine Diseases, Centre for Head and Orthopaedics, Rigshospitalet, Glostrup, Denmark; 5 Department of Occupational Therapy and Physiotherapy, Department of Clinical Medicine, Aalborg University Hospital, Aalborg, Denmark; 6 Department of Health Science and Technology, Aalborg University, Aalborg, Denmark

## Abstract

**Importance:**

Corticosteroid injections and exercise therapy are commonly used to treat chronic midportion Achilles tendinopathy, but the evidence for this combination is limited.

**Objective:**

To investigate the effect of corticosteroid injection and exercise therapy compared with placebo injection and exercise therapy for patients with Achilles tendinopathy.

**Design, Setting, and Participants:**

This was a participant-blinded, physician-blinded, and assessor-blinded randomized clinical trial of patients with Achilles tendinopathy verified by ultrasonography. Assessment of pain and function were conducted at baseline and at 1, 2, 3, 6, 12, and 24 months. Patients were recruited from a university medical clinic and a private rheumatology clinic in Denmark between April 2016 and September 2018. Data analysis was performed from June to September 2021.

**Interventions:**

Corticosteroid injection and placebo injection were performed with ultrasonography guidance. Exercise therapy was based on previous trials and consisted of 3 exercises done every second day.

**Main Outcomes and Measures:**

The primary outcome was the Victorian Institute of Sports Assessment–Achilles (VISA-A) score (range, 1-100, with 100 representing no symptoms) at 6 months. Secondary outcomes included pain measured using a 100-mm Visual Analog Scale for morning pain and pain during exercise (with higher scores indicating worse pain), global assessment (Likert scale), and tendon thickness.

**Results:**

A total of 100 patients were included, with 52 randomized to placebo (mean age, 46 years [95% CI, 44-48 years]; 32 men [62%]) and 48 randomized to corticosteroid injection (mean age, 47 years [95% CI, 45-49 years]; 28 men [58%]). Patients in the 2 groups had similar height (mean [SD], 177 [8] cm), weight (mean [SD], 79 [12] kg), and VISA-A score (mean [SD], 46 [18]) at baseline. The group receiving exercise therapy combined with corticosteroid injections had a 17.7-point (95% CI, 8.4-27.0 points; *P* < .001) larger improvement in VISA-A score compared with patients receiving exercise therapy combined with placebo injections at 6 months. No severe adverse events were observed in either group, and there was no deterioration in the long term (2-year follow-up).

**Conclusions and Relevance:**

Corticosteroid injections combined with exercise therapy were associated with better outcomes in the treatment of Achilles tendinopathy compared with placebo injections and exercise therapy. A combination of exercise therapy and corticosteroid injection should be considered in the management of long-standing Achilles tendinopathy.

**Trial Registration:**

ClinicalTrials.gov Identifier: NCT02580630

## Key Points

**Question:**

Compared with placebo injections, do ultrasonography-guided corticosteroid injections improve outcomes in adults with Achilles tendinopathy managed with exercise therapy?

**Findings:**

In this randomized clinical trial of 100 patients, statistically and clinically relevant improvement was found in the group receiving exercise therapy combined with corticosteroid injections.

**Meaning:**

In this study, a combination of exercise therapy and corticosteroid injection was more effective than exercise therapy and a placebo injection in the treatment of patients with long-standing Achilles tendinopathy.

## Teaser

This randomized clinical trial investigates the effect of ultrasonography-guided corticosteroid injection and exercise therapy compared with placebo injection and exercise therapy during a 3-month intervention in patients with Achilles tendinopathy.

## Introduction

Achilles tendinopathy (AT) is a common and often persistent overuse injury, especially in a sports-active population.^1,2^ It is characterized by local tenderness and swelling at the Achilles tendon, which affect an individual’s ability to participate in sport and leisure time activities. The prevalence and incidence rate have been reported to be 2.35 and 2.16 cases per 1000 person-years, respectively, in general practice.^3^ The lifetime incidence is 50% among elite endurance runners and 6% among sedentary people.^1,2^ Many patients with AT will seek health care, and it is one of the most common tendinopathies in the primary care sector.^4,5^

The most recent systematic review and network meta-analysis^6^ supports the use of exercise therapy in the management of chronic AT, and the effect is superior compared with wait-and-see or passive treatment modalities. Exercise therapy often includes 12 weeks of exercises performed 3 to 4 times per week. The clinical outcomes on pain and function take considerable time to manifest, with a gradual improvement over several weeks to months; however, not all patients recover fully, and residual symptoms often persist. Another treatment option is corticosteroid injection, which as a monotherapy has a documented good short-term clinical outcome in AT^7^ but not in the long term.^7,8^ A review^9^ from 2015 concluded that there was insufficient evidence to support the routine use of injection therapy in AT and recommended that placebo-controlled randomized clinical trials be conducted.

Preliminary evidence from a single cohort study^10^ indicates that exercise therapy may be combined with corticosteroid injections to achieve both good short-term and long-term outcomes. The data from the cohort,^10^ which included 93 consecutive patients, showed that at 6 months 42 patients were symptom free, 29 patients reported good results, and 16 were slightly improved. Overall, 94% of patients improved,^10^ highlighting the potential of this combination. The aim of the present randomized clinical trial was to compare exercise therapy and corticosteroid injections with exercise therapy and placebo injections in patients with AT as measured by the patient-reported outcome Victorian Institute of Sport Assessment–Achilles (VISA-A) score after 6 months. We hypothesized that exercise therapy and corticosteroid injections would be associated with larger improvements on the VISA-A compared with exercise therapy and placebo injections.

## Methods

### Study Design

This was a 2-group, 2-site trial and a patient-blinded, investigator-blinded, and assessor-blinded, parallel-group, placebo-controlled randomized superiority trial (see study protocol in Supplement 1). It was approved by the Greater Copenhagen Ethical Committee. Patients were included from a university clinic (Institute of Sports Medicine, University of Copenhagen) and from a local private rheumatology clinic between April 2016 and September 2018. The reporting of the study complies with Consolidated Standards of Reporting Trials (CONSORT) reporting guideline.

### Patients

All consecutive patients fulfilling the eligibility criteria were offered participation. Inclusion criteria were as follows: (1) patient-reported insidious onset of pain in the Achilles tendon region that was aggravated by weight-bearing activities and was worse in the morning and/or during the initial phases of weight-bearing activities; (2) pain and swelling located 2 to 6 cm proximal to the Achilles tendon insertion; (3) musculoskeletal ultrasonography imaging of the Achilles tendon showing local thickening (anterior-posterior) of more than 7 mm or more than 20% larger compared with the asymptomatic side; (4) Achilles tendon pain for more than 3 months; (5) and age between 18 and 65 years.

**Figure 1.**
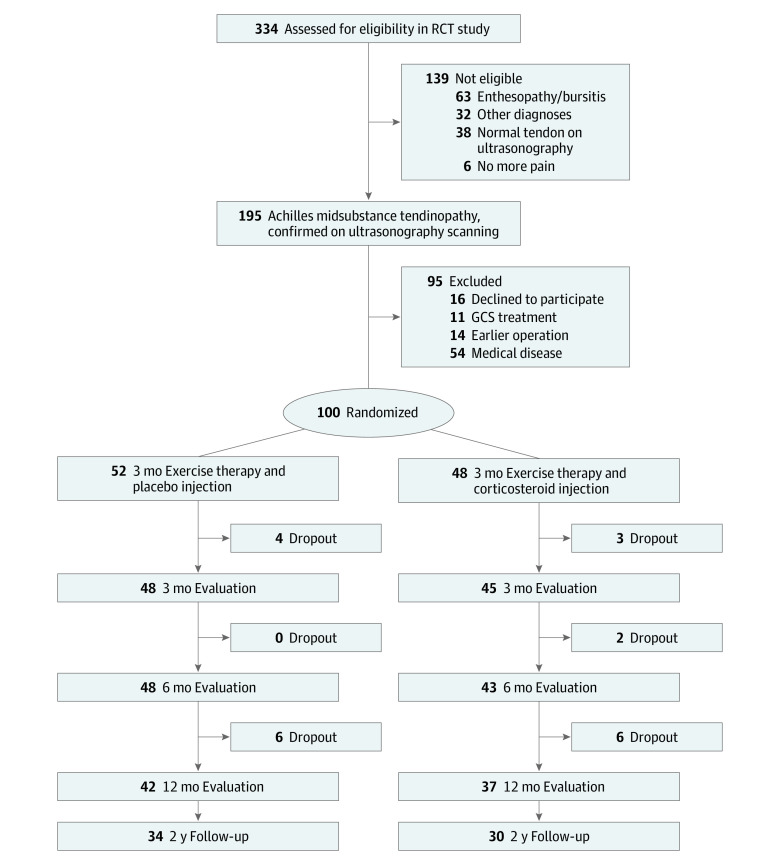
Participant Flow Diagram GCS indicates glucocorticosteroid; RCT, randomized clinical trial.

Exclusion criteria were as follows: (1) previous Achilles tendon surgery in the symptomatic lower limb; (2) known medical diseases (eg, diabetes or inflammatory rheumatic diseases); (3) previous corticosteroid injection for Achilles tendon pain within the past 6 months; (4) previous Achilles tendon rupture in the symptomatic lower limb; (5) body mass index (weight in kilograms divided by height in meters squared) greater than 30; (6) pregnant or planning pregnancy; and (7) injury or abnormality of the foot, knee, hip, and/or back or any condition that may interfere with participation in the study. We examined 317 patients, and 153 did not fulfill the inclusion criteria, mainly because of other diagnoses (involvement of other tendons, heel fat pad, plantar fascia, or neuropathic pain), especially enthesopathy-bursitis (63 patients). An additional 64 patients were excluded for other reasons mainly related to other abnormalities or medical diseases that interfered with the treatment protocol. Details are depicted in Figure 1.

### Randomization and Masking

After providing written informed consent, the patients were randomly assigned to 1 of 2 groups with a 1:1 allocation as per a computer-generated randomization schedule (MinimPy version 2010; M. Saghaei) using permuted blocks of random sizes (2 to 6). The primary investigator (F.J.), assessors (F.J., J.L.O., and T.F.O.), and administrator (not a coauthor of this article) of the randomization procedure were blinded to block sizes to ensure allocation concealment. After recruitment and baseline measurements, an independent office employee at the department (not a coauthor of this article) administered the allocation procedure and gave all patients consecutive research numbers. The allocation sequence was stored on a computer drive with exclusive access only for the responsible office employee. Only this employee and the pharmacist (not a coauthor of this article) who prepared the solution for injection were aware of the allocation group but did not have any contact with the patient during treatment and assessments.

Three medical practitioners (F.J., J.L.O., and T.F.O.) with a specialization in rheumatology performed the baseline and follow-up assessments. They were blinded to the content of the injection because the 2 types of injections were visually identical and had similar viscosity and the placebo injection was added with lipid emulsion for intravenous nutrition. Furthermore, the syringe was enclosed by nontransparent tape only showing the patient’s research number. The blinding was maintained until the last patient completed the last follow-up assessment, and the statistical calculation was performed. Data entry and the statistical analysis were performed blinded by independent investigators (A.S.J., M.S.R., and P.S.M.) not involved in the patient treatment and without knowledge of the treatment groups.

### Procedures

Patients in both groups had appointments with a physician (F.J., J.L.O., or T.F.O.) at study entry and at 1, 2, 3, 6, 12, and 24 months. The first 3 months were defined as the intervention period, when exercise therapy and the injections (both corticosteroid and placebo) took place. The patients were asked to refrain from jumping activity and running for the first 3 months. After the 3 months, they were encouraged to slowly return to their normal sports participation with Achilles tendon pain guiding their progression.

### Interventions

#### Injection

The syringe for injection was prepared by a pharmacist before patient arrival and put in a refrigerator marked with the patient’s research number. Before each injection, the physician made a routine clinical examination for any contraindications. At the first treatment visit, all patients were injected with either (1) 1 mL of methylprednisolone acetate (40 mg/mL) and 1 mL of lidocaine (10 mg/mL) (corticosteroid injection) or (2) 1 mL of lipid emulsion and 1 mL of lidocaine (10 mg/mL) (placebo injection).

Patients were offered up to 3 injections with an interval of at least 4 weeks between each injection. The second and third injections were given only if the patient reported morning pain greater than 20 mm on the 100-mm Visual Analog Scale for Pain (VAS; higher scores indicated worse pain) and/or pain during training greater than 40 mm on the VAS and/or self-reported satisfaction less than 3 on a scale of −5 to 5, where 5 is completely cured. Both types of injections were ultrasonography guided and placed peritendinous anterior to the tendon in the Kager triangle as close as possible to the thickest part of the tendon, or, in the case of neovascularization, as close as possible to the intratendinous vessel(s).

#### Exercise Therapy

The exercise therapy consisted of a heavy slow resistance program used in previous trials.^11^ The program should be performed 3 times per week using standard resistance equipment. Each session consisted of 3 two-legged exercises: (1) heel rises with bended knee in the seated leg press machine, (2) heel rises with straight knee in the leg press machine, and (3) heel rises with straight knee standing on a disc weight with the forefoot and with a barbell on the shoulders. The patients completed 3 or 4 sets for each exercise with a 2- to 3-minute rest between sets and a 5-minute rest period between the 3 different exercises. The number of repetitions decreased and the load gradually increased every week, guided by patient tolerance. The repetitions and loads were as follows: 3 times, 15-repetition maximum, in week 1; 3 times, 12-repetition maximum, in weeks 2 to 3; 4 times, 10-repetition maximum, in weeks 4 to 5; 4 times, 8-repetition maximum, in weeks 6 to 8; and 3 to 4 times, 6-repetition maximum, in weeks 9 to 12. All training sessions were documented in a training diary.

### Outcome Measures

#### Primary Outcome

The primary outcome measure was the change in the total score of the VISA-A questionnaire (range, 1-100, with 100 representing no symptoms) at 6 months compared with baseline, with additional secondary end points in VISA-A at other time points (1, 2, 3, 12, and 24 months). The VISA-A questionnaire evaluates 3 domains that are clinically relevant to patients: pain, function, and activity. The VISA-A questionnaire has been validated (construct validity), and shows good test-retest reliability in patients with AT.^12^

#### Secondary Outcomes

Secondary outcome measures that were collected at all time points were morning pain on a 100-mm VAS, pain during activity on a 100-mm VAS, ultrasonography measurement of the anterior-posterior thickness of the Achilles tendon described in a reliability study,^13^ and color Doppler flow in the Achilles tendon rated 0 to 3 according to the Newman grading scale.^14^ Patient-rated measures of return to sports participation were obtained, as well as self-reported Global Rating of Change on a Likert scale from −5 to 5, where 0 was unchanged from entry and 5 was completely cured.

### Adverse Events

All adverse events, defined as any negative or unwanted reaction to the intervention, was recorded at each physician visit, with special focus on fat atrophy, skin depigmentation, infections, and tendon ruptures. Furthermore, during the intervention period, the patients were asked to register any adverse events in their patient diary, including pain beyond a few days after the injections.

### Statistical Analysis

Data analysis was performed from June to September 2021. There is no established minimal clinically important difference for the VISA-A score for midportion AT,^15^ but the minimal clinically important difference for insertion AT is 6 points.^16^ This study was powered to detect a between-group difference in the VISA-A score of 10 points at the primary end point at 6 months. On the basis of scores in a previous study,^11^ a type I error of 5%, and a type II error of 20% (80% power), it was calculated that 47 patients were needed in each study group. To allow for a 5% potential dropout and a potentially larger SD than previous trials, we increased the sample size to 50 patients per group and included a total of 100 patients. The intention-to-treat principle was used for all analyses.

Analyses were conducted on all outcome measures at entry and at 1, 2, 3, 6, and 12 months. A 2-year follow up was planned, but was extended for practical reasons mainly because of the COVID-19 pandemic. Between-group comparisons of treatment effect for all primary and secondary outcomes were performed with a mixed-effects model with patient as a random effect, and time of assessment, study group, and baseline values of the outcome as fixed effects. To assess for superiority, mean between-group differences in changes from baseline and 2-sided 95% CIs were calculated. Q-Q plots were visually examined to ensure that the assumption of normal distribution was not violated prior to conducting the mixed model analysis. Doppler score was analyzed with nonparametric Mann-Whitney *U* tests. Significance was set at 2-sided *P* < .05. Data were analyzed with GraphPad statistical software version 9.3.1 (Prism).

## Results

Participants were recruited from April 21, 2016, to September 13, 2018. A total of 100 patients were enrolled and randomized, 52 to exercise therapy and placebo injection (mean age, 46 years [95% CI, 44-48 years]; 32 men [62%]) and 48 to exercise therapy and corticosteroid injection (mean age, 47 years [95% CI, 45-49 years] years; 28 men [58%]). The 2 treatment groups had similar height (mean [SD], 177 [8] cm), weight (mean [SD], 79 [12] kg), and VISA-A score (mean [SD], 46 [18]) at baseline. The baseline characteristics of the 100 patients are presented in Table 1. In the corticosteroid group, 12 patients received 3 injections, 18 patients received 2 injections, and 15 patients received 1 injection, excluding 3 dropouts. In the placebo group, 35 patients received 3 injections, 10 patients received 2 injections, and 3 patients received 1 injection. All but 1 patient complied with the recommendation to refrain from running and jumping in the intervention period.

**Table 1.** Participant Characteristics at Baseline

Characteristic	Mean (95% CI)^a^	
	Placebo injection (n = 52)	Corticosteroid injection (n = 48)
Age, y	46 (44-48)	47 (45-49)
Sex, No. (%), participants		
Male	32 (62)	28 (58)
Female	20 (38)	20 (42)
Height, cm	178 (176-180)	175 (173-177)
Weight, kg	81.4 (78.2-84.6)	76.3 (72.9-79.7)
Body mass index^b^	25.7 (25.0-26.4)	24.8 (24.0-25.6)
Symptom duration, mo	20 (14-26)	26 (16-36)
Bilateral symptoms, No. of participants	15	14
Achilles tendon thickness, mm	8.8 (8.4-9.2)	8.6 (8.2-9.1)
Doppler ultrasonography score (0-3)	1.62 (1.43-1.81)	1.84 (1.66-2.02)
Morning pain (VAS score range, 0-100 mm)^c^	41 (34-48)	40 (33-47)
Pain at activity (VAS score range, 0-100 mm)^c^	48 (41-55)	45 (38-52)
VISA-A score (range, 0-100)^d^	50 (46-54)	43 (38-48)

Abbreviations: VAS, Visual Analog Scale; VISA-A, Victorian Institute of Sport Assessment–Achilles.

^a^ There were no differences between groups for any parameters at baseline.

^b^ Body mass index is calculated as weight in kilograms divided by height in meters squared.

^c^ On the VAS, higher scores indicate worse pain.

^d^ On the VISA-A, a score of 100 represents no symptoms.

There were no between-group differences in the dropout rate. Ten patients (10%) dropped out before the 6-month primary end point. Four patients did not start treatment after randomization, 1 patient changed residence, and 5 patients were unable to train. Furthermore, 11 patients (6 in the placebo group and 5 in the corticosteroid group) dropped out (total, 21%) at 12 months, mainly because they were seeking other treatments. The planned 2-year follow-up was performed at 24.7 months after entry for the placebo group and at 24.8 months after entry for the corticosteroid group (range, 22-32 months).

### Primary Outcome: VISA-A Score

**Figure 2.**
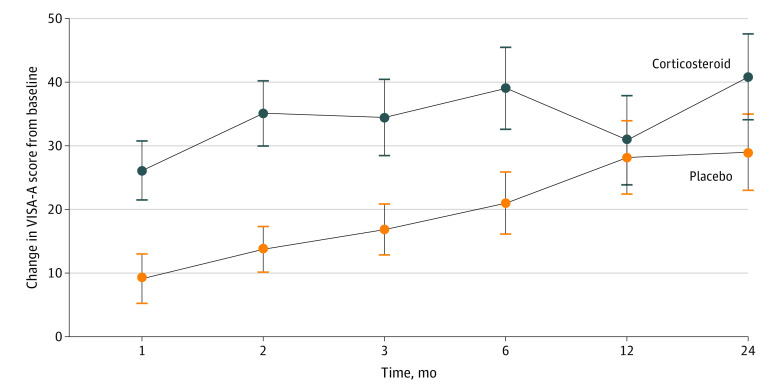
Primary Outcome The figure illustrates the primary outcome, improvement in Victorian Institute of Sports Assessment–Achilles (VISA-A) score from baseline to 6 months and the secondary outcomes of the VISA-A score at other time points (1, 2, 3, 12, and 24 months). Error bars indicate the 95% CIs. Compared with the placebo group, the group randomized to corticosteroid injection and exercise therapy had a 17.7-point (95% CI, 8.4-27.0 points; *P* < .001) larger improvement in VISA-A score from baseline to 6-month follow-up. The group receiving corticosteroid injection had a significantly larger improvement vs the placebo group across all time points except the 12-month follow-up (all *P* < .001).

The primary analysis showed that the group randomized to corticosteroid injection and exercise therapy had a 17.7-point (95% CI, 8.4-27.0 points; *P* < .001) larger improvement in VISA-A score from baseline to 6 months follow-up compared with the placebo group (Figure 2). The group receiving corticosteroid injection had a significantly larger improvement across all time points except the 12-month follow-up. Both groups improved over time. Table 2 depicts the exact scores at any time point.

**Table 2.** Exact Scores for Both Groups at Any Time Point

Score and group	Mean (95% CI)						
	Entry	1 mo	2 mo	3 mo	6 mo	12 mo	24 mo
VISA-A score (range, 0-100)^a^							
Corticosteroid	43 (38-48)	68 (64-72)	78 (74-82)	77 (72-82)	82 (77-87)	76 (70-82)	86 (82-90)
Placebo	50 (46-54)	60 (55-65)	65 (61-69)	68 (63-72)	72 (67-77)	79 (73-85)	82 (76-88)
VAS morning pain score (range, 0-100 mm)^b^							
Corticosteroid	40 (33-47)	16 (10-22)	6 (2-10)	7 (3-11)	12 (7-17)	20 (12-28)	9 (3-15)
Placebo	41 (34-48)	29 (21-37)	23 (16-30)	16 (10-22)	11 (5-17)	12 (7-17)	8 (3-13)
VAS function pain score (range, 0-100 mm)							
Corticosteroid	45 (38-52)	20 (14-26)	13 (8-18)	10 (5-15)	13 (7-19)	19 (12-26)	10 (4-16)
Placebo	48 (41-55)	23 (16-30)	21 (14-28)	16 (10-22)	12 (7-17)	12 (7-17)	6 (2-10)
Ultrasonography-measured thickness, mm							
Corticosteroid	8.6 (8.2-9.1)	6.7 (6.3-7.1)	6.2 (5.9-6.6)	6.0 (5.6-6.3)	6.7 (6.2-7.3)	7.7 (7.1-8.3)	7.1 (6.4-7.8)
Placebo	8.8 (8.4-9.2)	8.5 (8.1-9.0)	8.4 (8.0-8.9)	8.2 (7.8-8.7)	7.8 (7.4-8.2)	7.4 (6.9-7.8)	6.9 (6.4-7.4)

Abbreviations: VAS, Visual Analog Scale; VISA-A, Victorian Institute of Sport Assessment–Achilles.

^a^ On the VISA-A, 100 represents no symptoms.

^b^ On the VAS, higher scores indicate worse pain.

### Secondary Outcomes

**Figure 3.**
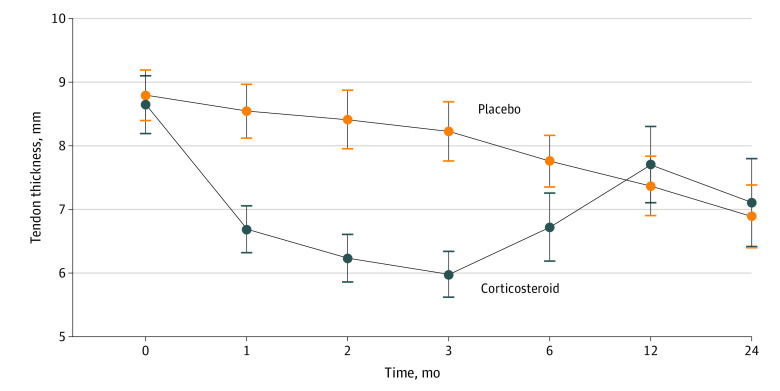
Tendon Thickness The figure illustrates the ultrasonography-measured thickness of the Achilles tendon. Error bars indicate the 95% CI. Thickness of the Achilles tendon improved significantly more in the corticosteroid group compared with the placebo group at 1 month (mean difference, 1.9 mm; 95% CI, 1.1-2.6 mm; *P* < .001), at 2 months (mean difference, 2.2 mm; 95% CI, 1.4-3.0 mm; *P* < .001), at 3 months (mean difference, 2.2 mm; 95% CI, 1.5-3.0 mm; *P* < .001), and at 6 months (mean difference, 1.0 mm; 95% CI, 0.1-1.9 mm; *P* = .02).

Ultrasonography-measured thickness of the Achilles tendon improved significantly more in the corticosteroid group compared with the placebo group at 1 month (mean difference, 1.9 mm; 95% CI, 1.1-2.6 mm; *P* < .001), at 2 months (mean difference, 2.2 mm; 95% CI, 1.4-3.0 mm; *P* < .001), at 3 months (mean difference, 2.2 mm; 95% CI, 1.5-3.0 mm; *P* < .001) and at 6 months (mean difference, 1.0 mm; 95% CI, 0.1-1.9 mm; *P* = .02) (Figure 3). The Doppler ultrasonography score was lower in the corticosteroid group at 1 month (Mann-Whitney *U*, 454; *P* < .001), 2 months (Mann-Whitney *U*, 234; *P* < .001), 3 months (Mann-Whitney *U*, 263; *P* < .0001), and 6 months (Mann-Whitney *U*, 653; *P* < .01), but not at baseline or 12 and 24 months. For morning pain on the VAS, there was a significantly larger improvement of 15.6 mm (95% CI, 2.6-28.6 mm; *P* = .01) after 2 months in the group randomized to corticosteroid injection. For pain during activity on the VAS, we could not demonstrate any significant differences at any time points. Patients’ overall assessment of the treatment effect was in favor of corticosteroids, with significant differences at 1 month (mean difference, 0.8; 95% CI, 0.1-1.6; *P* = .02), 2 months (mean difference, 1.8; 95% CI, 1.0-2.6; *P* < .001), and 3 months (mean difference, 1.3; 95% CI, 0.5-2.2; *P* < .001), whereas no difference was observed at 6, 12, and 24 months (secondary outcomes are presented in eTables 1-3 and eFigures 1-3 in Supplement 2).

### Adverse Events

A total of 215 injections were administered (87 corticosteroid and 128 placebo). Injection pain was indicated in the patients’ diaries, with a mean (SD) pain score of 12 (14) of 100 in the corticosteroid group and 14 (17) of 100 in the placebo group, with no significant differences between groups. No severe adverse events (eg, infection, tendon rupture, subcutaneous depigmentation, or atrophy) were recorded in either of the groups.

## Discussion

To our knowledge, this randomized clinical trial is the first study to directly investigate the possible additive effect of a corticosteroid injection to exercise therapy when compared with a placebo injection and exercise therapy in patients with AT. This trial found a significant and clinically relevant effect at the primary end point at 6 months. In general, we observed larger improvements in the group receiving corticosteroid injection across the entire follow-up period. Importantly, we did not observe any detrimental effect of corticosteroid on clinical outcomes in the later phase (12-24 months after treatment start). These findings suggest that corticosteroid injection is effective when combined with exercise therapy in patients with long-standing AT and without major adverse events. In the present study, we used the VISA-A score, which is the most commonly used patient-reported outcome measure for trials investigating AT treatment. Other studies have found the VISA-A score to be valid and reliable, with a test-retest intraclass correlation coefficient 0.99 and a SE of the measurement 2.53, and the minimal detectable change at the 95% confidence level was 7 points.^17^ It should be noted that a recent psychometric analysis indicated that the construct and content validity of the VISA-A score is in dispute.^18^ However, as this score is the only currently available patient-reported outcome measure and thus allows for comparisons to other existing studies, we have used it for the current study.

### Explanation of Findings

The present study adds to the current knowledge and suggests that corticosteroid injection can play a valuable role in the management of long-standing AT when combined with exercise therapy. Exercise therapy is hypothesized to work by activating mechanotransduction pathways within the extracellular matrix that influence the anabolic and catabolic responses of the tissue, which leads to positive tissue adaptations and a reduction in pain.^11^ The mechanism behind corticosteroids in AT is currently unclear; however, corticosteroids are known to inhibit cell proliferation^19^ and a general fibroblast stimulation in tendon tissue.^20^ More lately, circadian rhythm has been demonstrated to play a major role in the dynamic turnover of extracellular matrix in tendon^21^ and is not directly regulated by exercise itself.^22^ Thus, exercise and corticosteroids treatments may act through 2 different pathways that could potentially have an additive effect.

### Comparison With Previous Findings

Previous trials have investigated different types of injections for AT, including monotherapy and when combined with, for example, exercise therapy. A recent network meta-analysis^6^ on all trials investigating injection therapy for AT combined many types of injections (platelet-rich plasma, corticosteroid, high-volume injections, and autologous blood) found that exercise therapy was comparable to injection therapy for midportion tendinopathy. It has also been shown that platelet-rich plasma is not effective compared with placebo injection.^23^ This finding suggests that some types of injections (eg, platelet-rich plasma) are unlikely to be effective treatment options for AT, whereas our study suggests that corticosteroid injection is effective when combined with exercise therapy.

In a systematic review^8^ of corticosteroid injections as treatment for tendinopathy, good short-term (4-8 weeks) but poor intermediate (13-26 weeks) and long-term (≥52 weeks) outcomes were reported. However, most of the included studies were of patients with lateral epicondylitis (ie, a non–weight-bearing tendon). Only 1 study^7^ included patients with AT treated with 3 corticosteroid injections as monotherapy followed by unrestricted rehabilitation. Importantly, in the present study, all patients were recommended to refrain from all running and jumping activities in the first 3 months (99% complied), and all patients followed a progressive exercise therapy. This could explain the superior effect of corticosteroid injections in both the short and intermediate term (1-6 months), as well as the continued good results in the long term (≥1 year) in our study. However, these findings may not necessarily apply to all tendons since corticosteroid injection has not provided an additive effect to exercises for shoulder pain^24^ or lateral epicondylitis,^25^ whereas it has been suggested to have an additive effect in plantar fasciitis.^26^ In our study, no severe adverse events associated with the corticosteroid were registered, and the injections were almost pain free. Tendon ruptures after corticosteroid injections are often described in case studies,^27^ but in controlled studies only 1 Achilles tendon rupture has previously been reported after 3 corticosteroid injections in patients with AT who underwent aggressive rehabilitation that included running after only a few days.^7^ It should be noted that the reduction in tendon size with corticosteroid injection represented a normalization upon a tendinopathy-enlarged tendon, not tendon atrophy. In our study, the patients refrained from running and jumping activities during the first 3 months, but started exercise therapy a few days after the injection.

### Limitations

This study has limitations that should be addressed. We used the VISA-A score, which is the most commonly used patient-reported outcome measure for trials investigating AT treatment. However, a recent psychometric analysis^18^ indicates that its construct and content validity are in dispute.

## Conclusions

Corticosteroid injections combined with exercise therapy are associated with larger improvement in patient-reported outcomes compared with placebo injections combined with exercise therapy. The effect is visible at both short-term and long-term follow-up (up to 24 months) and is not associated with a higher rate of adverse events compared with placebo injections.

## Notes

Supplement 1.
Trial Protocol and Statistical Analysis Plan

Supplement 2. **eFigure 1.** Secondary Outcome: Improvement in VAS-Morning Pain From Baseline to 1, 2, 3, 6, 12, and 24 Months **eFigure 2.** Secondary Outcome: Improvement in VAS-Activity Pain From Baseline to 1, 2, 3, 6, 12, and 24 Months **eFigure 3.** Secondary Outcome: Likert Score at All Time Points **eTable 1.** Likert Score (-5 to +5) Divided in Groups at All Time Points **eTable 2.** Likert Score: Number of Patients Much Improved or Cured at All Time Points **eTable 3.** Likert Score: Mean and 95% CI at All Time Points

Supplement 3.
Data Sharing Statement
