# Supplementary material for: Effect of Ultrasonography-Guided Corticosteroid Injection vs Placebo Added to Exercise Therapy for Achilles Tendinopathy: A Randomized Clinical Trial
Source: JAMA Netw Open. 2022 Jul 11;5(7):e2219661. doi: 10.1001/jamanetworkopen.2022.19661 (PMC9274322; doi:10.1001/jamanetworkopen.2022.19661)
Supplement: Supplement 1. — Trial Protocol and Statistical Analysis Plan [file jamanetwopen-e2219661-s001.pdf]

Ethical approved protocol version 3. Original in danish.

Protocol title: Heavy weight training as a treatment for Achilles tendinopathy supplemented with injection of glucocorticosteroid or local anesthetics.
Short title: Exercise and injection treatment for Achilles tendinopathy.

Project coordinators: Jens Lykkegaard Olesen and Finn Johannsen
Department of Sports Medicine
Bispebjerg Hospital

Sponsor: ISMC
Institute of Sports Medicine Copenhagen

1. Purpose

to investigate whether treatment with heavy strength training in combination with injection of local glucocorticosteroid reduces pain and improves function more than treatment consisting of heavy strength training with injection of anesthetics in patients with Achilles tendinopathy.

2. Place of project execution

Bispebjerg Hospital, Department of Sports Medicine
with two primary investigators: Chief Physician Jens Lykkegaard Olesen and Chief Physician Finn Johannsen.
The study will include patients from the Department of Sports Medicine, Bispebjerg Hospital, Bispebjerg Bakke 23, entrance 8 1st floor and the Furesø rheumatologists.

3. Researchers and author order

- Jens L Olesen1, Finn Johannsen1*, Tommy Øhlenschläger1, Michael Rathleff2, Michael Kjær1.
- Investigators, clinically responsible: Jens Lykkegaard Olesen (JLO), chief physician Ph.D.
Chief physician Finn Johannsen.

* shared first authorship
1: Department of Sports Medicine (ISCM), Bispebjerg Hospital
2: Center for Sensorimotor Interaction, Aalborg University

4. Sponsor

The trial is sponsored by the Department of Sports Medicine, Bispebjerg Hospital.

5. Background to the study:

Achilles tendinopathy is a frequent and often long lasting disease, especially in sports. The cumulative incidence in former elite athletes is 50% where by comparison it is 5.9% in inactive (Kujala, 2005). In particular, athletes who involve running and jumping are at risk (Kvist 1991). When running, the incidence is 9% (Lysholm 1987).
As primary treatment, eccentric exercises are recommended, where improvement of between 60% to 90% has been described (Silbernagel, Maffuli, 2008, 2010, Ohberg). Other studies have shown the effect of stretching exercises (Porter 2002) and in a randomized study, no difference in outcome could be seen with treatment with eccentric exercises compared with stretching exercises (Nørregård 2007). In a recent study at our institute, heavy slow strength training has also been shown to be equivalent in treatment effect compared to eccentric training (Beyer R in prep).
Glucocorticoid injection (GCS) is often used in the clinic, but as there are rarely signs of inflammation in the Achilles tendons, the rationale behind

the treatment is controversial (Khan 2002). However, a randomized clinical trial (RCT) has shown good effect of GCS injections given ultrasound-guided in chronic Achilles tendinopathy (Fredberg 2004). A significant short-term effect was seen here on symptoms and the thickness of the tendons, but relapse of tendinopathy after 6 months possibly due to an aggressive rehabilitation program. In exercise RCT, 60 to 90% are seen to have an effect of the treatment, but in a pragmatic clinical trial it has been observed that only 26% have a satisfactory effect of exercise alone (Wetke in press 2014). Likewise, only 10% had an effect when using a program consisting of eccentric exercises for home training (Ram 2013). That is, exercise alone does not solve the problem for everyone, but how do we treat the rest? The study by Wetke et al found a good effect of supplementing the training with injection of glucocorticosteroid mixed with local anesthetics, but whether this effect was due to a pain-reducing effect of the local anesthetic or an anti-inflammatory effect of glucocorticosteroid is not known. To date, no RCT study has been performed looking at the combined effect of exercise and injections. Our hypothesis is that training combined with GCS will have a better effect than training combined with injection of local anesthetics.
The existing standard treatment for Achilles tendinopathy is in accordance with clinical guidelines in the Danish Sports Medicine Society (DIMS) and in the Danish Rheumatological Society (DRS) primarily load reduction supplemented with training with stimulating exercises. If necessary, this can be supplemented with injection of glucocorticosteroid, usually mixed with local anesthetics.

The purpose of our study is to investigate whether the existing standard treatment with load reduction with reduced running and jumping activities and slow training with slow heavy strength training in combination with injection of glucocorticosteroid reduces pain and improves function more than treatment consisting of the same training and analgesic injection .

New knowledge about other tendinopaties has shown that even with a combination of guidance and glucocorticoid injection, many have continued long-term symptoms after 2 years. This has not been well studied for Achilles tendinopathy with combination therapy.

Our study is based on the following hypothesis:

Treatment with slow heavy strength training and reduced running and jumping activities combined with injection of glucocorticosteroid (current standard treatment) provides better pain reduction and increase in functional level than treatment consisting of the same load reduction, training and injection with local anesthetics measured on VISA-A.

6. Material and method

Patients with suspected Achilles tendinopathy are usually referred to the sports medicine department BBH or specialist practice in rheumatology. When the center receives a referral with suspected Achilles tendinopathy, the patient will be contacted and oral information about the trial will be provided over the phone. The person is subsequently asked if he or she would like to participate. If persons are positive about participating in the trial, the person will be summoned through the secretary. If patients do not wish to participate in the trial, they will be offered regular treatment.

Inclusion criteria

1. Ultrasound diagnosed middle Achilles tendinopathy.
2. In the case of unilateral symptoms, symptomatic Achilles tendons should be more than 20% thicker than on the asymptomatic side or more than 7 mm thick
3. In the case of bilateral symptoms, the Achilles tendon must have a diameter of more than 7 mm.
4. Duration of pain should be a minimum of 3 months

5. Participant is a minimum of 18 years and a maximum of 65 years.
6. Participant may give relevant and adequate, informed consent.

Exclusion criteria.
1. Previous surgery of the lower extremity. Exceptions are Arthroscopy and cartilage resections in the knee.
2. No known medical conditions, including insulin-requiring diabetes mellitus or rheumatic diseases.
3. Infection of the foot or lower leg.
4. Mental state that does not allow participation.
5. Not considered able to follow the training intervention.
6. Absence of presence in the region during the project period.
7. Daily use of painkillers.
8. Can not read or understand Danish *.
9. Steroid injection for the treatment of Achilles tendinopathy within the last 6 months **.
10.Previous allergic reaction to treatment with steroid (Depomedrol) or topical anesthetics.
11. Pregnancy / lactation or planning of pregnancy during the intervention period.
12. BMI over 30.

* Due to the cost of an interpreter, the subject has to read and understand Danish.
** Due to mild discomfort with steroid injection around the Achilles tendon, we rely on the patient to remember if this has been done before. If the patient cannot remember when the injection was given, the patient must obtain information before inclusion

Patients are randomized into 2 groups, see chart below.

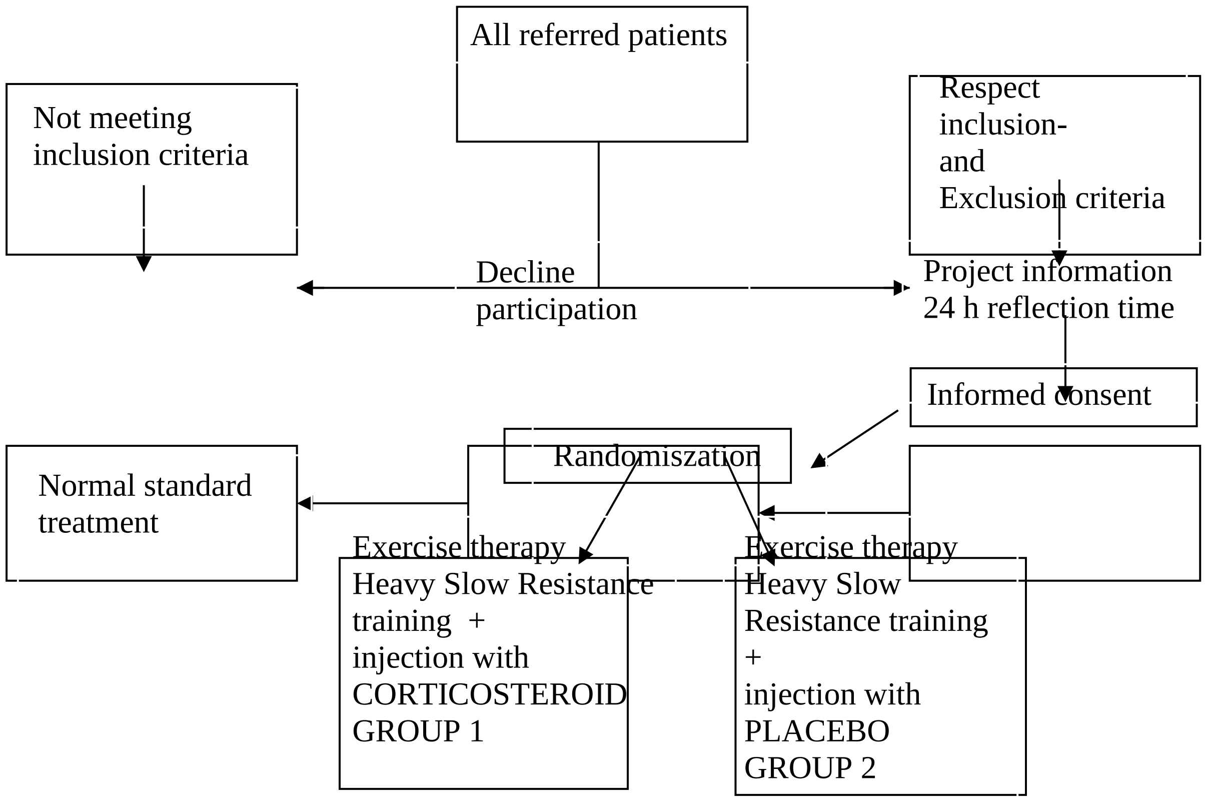

Randomization principle
The randomization must be preceded by informed consent and power of attorney. The randomization of patients is performed by an independent laboratory technician who is not involved in the patient treatment. This is done by computer randomization program. The patients will be evenly divided into 2 groups. The laboratory technician is then responsible for filling out randomization sheets and mixing injection for the investigator. The laboratory technician must prepare the 2 injections according to the description below, and must keep the randomization sheet for the patients secretly and securely locked until the end of the experiment, which is defined as a 24-month follow-up of patient number 100. The laboratory technician will also be responsible for used medication. The laboratory technician will not have contact with the patients.

Blinding
The study will be double-blind. The doctors who give the injections and examine the patients before and after treatment, as well as the physiotherapists who instruct and supervise the training, are blind to which injection is given. Likewise, patients are blinded to whether they are receiving one or the other injection.

The 2 treatment arms are:
1: Treatment with heavy slow resistance training, stretching exercises and reduced running and jumping activities combined with injection of local anesthetics. This consists of lidocaine as well as intralipid. Lidocaine is a mild analgesic and the intralipid is added so that the mixture is indistinguishable from Depomedrol. Detailed description of these can be found in the sections below.
2: Standard treatment consisting of heavy slow resistance training, stretching exercises and reduced running and jumping activities combined with glucocorticosteroid injection. This injection consists of Depo-medrol ® (methylprednisoneolone acetate) 1ml and lidocaine 10mg / ml 1ml. Detailed description of these can be found in the sections below.

Description of load reduction
For the first 3 months, patients should refrain from jumping and running activities (impact training). They are welcome to do non-impact training such as cycling, swimming, rowing and strength training. After 3 months, they must slowly return to their previous sports activity with jumping and running.

Description of heavy slow resistance training
Strength training will consist of a progressive training program with training 3 times a week starting with the physiotherapist 1 week after the injection. The training is supervised by a physiotherapist for the first 3 months with team training one day every 2 weeks. As the tissue becomes stronger and the pain becomes less, the tissue is challenged at higher loads. Instruction in the exercises will be handled by two project physiotherapists. The exercises are also handed out in text and pictures. In the case of bilateral symptoms, both sides must be trained, but only one side is included in the evaluation of effect parameters. In the case of unilateral symptoms, only the symptomatic side is trained.

Description of glucocorticosteroid injection
The standard treatment consists of the mixture of the glucocorticosteroid: Depomedrol 1 ml and Lidocaine 1%. A mixture of 1 ml Depomedrol and 1 ml Lidocaine 1% (10 mg / ml) will be made immediately before injection. 2 ml of the mixture between tendon and paratenon corresponding to the thickest site

will then be injected under ultrasound guidance. The mixtures will in both cases be performed by another person (laboratory technician) than the examiner, so that both the patient and the examiner are blinded. The project staff member responsible for the randomization and mixing of the syringes will also be responsible for completing and maintaining the medication list and maintaining the blindness of patients and caregivers. Whether the symptoms are bilateral or unilateral, only one side which at the time of inclusion was selected for treatment is treated. As long as the tendon is over 7 mm thick or more than 20% thicker than the healthy one given injection at inclusion and 1 and 2 months after inclusion, i.e. maximum 3 times. This is the usual standard treatment.

Description of injection of local anesthetics

Local anesthetic treatment will be made a mixture of 1 ml lidocaine 1% (10 mg / ml) and 1 ml 20% Intralipid. Intralipid will be added so that one cannot distinguish between local anesthetics and the steroid injection on the basis of altered appearance of the fluid. The same technique will be used as with steroid injection. Intralipid is not normally present at the centers and is purchased for the trial. The project employee will keep the accounts with consumption, as well as the date of opening the bottle with intralipid. Any residue will be destroyed after patient number 50 has received the last injection. The mixing will be performed by another person (laboratory technician) than the examiner, so that both the patient and the examiner are blinded. The mixture for injection is produced by the project worker immediately before use.

7. Experimental design

Double-blind randomized controlled trial with follow-up after 1,2, 3, 6, and 12 months as well as after a minimum of 24 months. 50 patients are included in each of the 2 groups. See section 11 for sample-size calculation. Primary endpoint is difference in VISA-A after 6 months.

8. Outcome measures

Primary outcome measures:

VISA-A score

Secondary power parameters:

Thickness of the Achilles tendon measured by ultrasound scan, cross-section of the tendon's thickest place.
Doppler activity graded from 0-3 according to Newmann's grading scale.

Immediately after inclusion, the following parameters are recorded and noted in the CRF:

Names and initials.
Date of birth and year of birth
Sex
Weight
Height
Uni- or bilateral heel pain, as well as an indication of which is included in the trial
Duration of pain
Formerly treatment in relation to the disorder
Taking NSAIDs and painkillers for current heel pain
Concomitant medication consumption in addition to medication for the current heel pain.

Diameter of the Achilles tendon measured by ultrasound scan across the thickest site.
Doppler activity (graded from 0-3 according to Newmann's grading scale).
Physical activity level measured as preferred sports activity as well as weekly time consumption before the injury occurred
VISA-A

At each physiotherapist training instructions patients are asked about completed training since the last and compliance is registered.
At each medical check-up the patients must indicate the VISA-A score and whether the injection itself hurt (VAS 0-100) and side effects are recorded in the following days after the injections with symptom and number of days.

9. Practical implementation of the study

The patient groups are included from the mentioned "places for project execution". Before patients are included in the trial, they must read “PARTICIPANT INFORMATION - GENERAL INFORMATION” and “PARTICIPANT INFORMATION - RIGHTS OF SUBJECTS IN A

BIOMEDICAL RESEARCH PROJECT” which deals with general information about being a subject in a biomedical experiment and what rights they have as a subject. In addition, subjects must read "PARTICIPANT INFORMATION - ABOUT THE PROCEEDINGS OF THE EXPERIMENT", which deals with information about the experiment and the course of the experiment. Guidelines for the submission of oral and written information follow this procedure:
1) If possible, the subject will be sent written material before arriving at the center.
2) The oral and written information is provided in an undisturbed environment by the investigator.
3) It must be stated that this is a request for participation in a scientific experiment.
4) It must be stated that the patient is examined 7-8 times, where each examination itself is expected to take 30 minutes. Patients are told that they are expected to participate in all examinations if they wish to participate.
5) A copy of both "PARTICIPANT INFORMATION - GENERAL INFORMATION" must be handed out to the patient.
6) The patient must be provided with "PARTICIPANT INFORMATION - RIGHTS OF SUBJECTS IN A BIOMEDICAL RESEARCH PROJECT" and the pre-decision of the Science Ethics Committee "Before you decide".
7) The participant must have up to 24 hours to decide whether he or she will participate in the project. The 24 hours are calculated after the subject has read the written information, and listened to the oral information and has had the opportunity to ask questions.
8) The trial participant receives a copy of the consent after signing by the trial leader and trial participant

The subjects must give consent before collecting information that will be used in the experiment. The consent must be given in writing after oral and written information about the project. Subjects must be informed that they can withdraw from the trial at any time without justification, and that they will then be offered regular treatment.

It is ensured that inclusion criteria are met and none of the exclusion criteria are met.
The patient is randomized to one of the 2 groups
Baseline data where the patient fills in primary as well as secondary effect parameters in CRF. In addition, the Achilles tendon is ultrasound scanned by the doctor and the diameter and flow are determined.
4. The patient receives the intervention to which they are randomized. After receiving the injection, the patient is sent 1 week later to a physiotherapist, who instructs in the training.
Both groups are checked by the doctor after 1 and 2 months. Here, the ultrasound scan and possibly the injection as well as VISA-A are repeated.
Both groups receive follow-up on an exercise program with a physiotherapist 1 day every 2 weeks for the first 3 months. This with regard to most appropriate progression of the weight load. Compliance with the training is registered by currently indicating how many days have been trained weekly since the last visit.
7. Both groups receive 3 months follow-up. Here VISA-A is repeated, and the diameter of the Achilles tendon + Doppler is measured by ultrasound scan.
8. Both groups receive 6 months follow-up. Here VISA-A is repeated and the diameter of the Achilles tendon + Doppler is measured by ultrasound scan. Questions are asked about activity level and compliance with the training.
9. Both groups receive 12 months follow-up. Here VISA-A is repeated and the diameter of the Achilles tendon is measured by ultrasound scan + Doppler. Questions are asked about activity level and compliance with the training.
10. Both groups receive long-term follow-up after a minimum of 24 months. Here VISA-A is repeated and the diameter of the Achilles tendon is measured by ultrasound scan + Doppler. Questions are asked about activity level and compliance with the training. Once the 24 month follow-up is complete, the patient is terminated in CRF and medical record. Since the 24 month measurement point has been added in relation to the original project, new participant information is provided
11. The code for the individual is broken after 24 months of follow-up by the last patient. Individuals who have not achieved pain relief are offered steroid injection, including those who were randomized to steroid injection during the trial.

The above calls have a margin of +/- 2 weeks without it being considered a protocol deviation.

11. Statistics

The risk of finding a difference between the groups, even though there is really none, is set at 5% (risk of type I error), while the risk of ignoring a difference between the groups, which is actually present, is set at 20% (type II error). The groups will be compared with an unpaired t-test. Significance level is set to 0.05.
The minimum relevant difference in VISA-A is set to 10. Based on other studies regarding pain intensity, we expect a mean value at baseline of 50 and after treatment 80. Standard deviation is estimated to be 18 on VISA-A. Power of 80% gives a sample size of 45 participants in each group. A total of 100 patients are expected to be examined in each group in case of data loss and absenteeism.
Data processing:
Data are entered in the CRF in a patient file, where all information from the individual subject is entered. Folders are stored in the departments mentioned under point 2 "place of execution of the project". After a 24-month follow-up has been done on patient number 50 in each group, 2 independent persons will enter data into Epidata, after which double validation will be

performed to ensure that the data has been entered correctly. This will take place on 2 independent computers both of which are password protected.

Analysis of data will take place in consultation with statisticians.
All data will be deleted 5 years after the end of the experiment, except that additional protocols are prepared for the experimental group for further data processing or that the projects are unexpectedly not completed.
All data on BBH internal H-drives will be backed up.

12. Ethics

The investigation complies with the conditions mentioned in the Declaration of Helsinki III. The entire trial protocol must be approved by the Science Ethics Committee of the Capital Region before the start of the trial. The project is also notified to the Danish Data Protection Agency and clinicaltrial.gov.
Generally, patients with this disorder are offered guidance in load reduction in the form of reduced exercise volume as well as exercises. The first 3 months the patient must stop jumping and running activities (impact training). They are welcome to do non-impact training such as cycling, swimming, rowing and strength training. After 3 months, they must slowly return to their previous sports activity with jumping and running. In addition, slow heavy strength training is recommended. This is often supplemented with glucocorticosteroid injection.
Half of the patients are treated in our study with local anesthetic injections. This is also safe as it has not yet been clarified that steroid injection is superior to injection with local anesthetic in the long run. A recent study by Kongsgaard et al shows that steroid injection for chronic tendon injury in the knee tendon has a good short-term effect, but poor long-term effect when compared to heavy strength training. It is therefore important to find out if steroid injection for this patient group increases the effect of heavy strength training. There are only a few active treatments that could be considered as a basis for comparison. These are shockwave and cross-massage by a physiotherapist. The value of these treatments has been debated and with regard to the use of shockwaves and cross-massage, the blindness towards the patients will not be maintained.

Depomedrol and Lidocaine injection are standard treatment at the sports medicine clinic and in specialist practice, and are recommended in both the DIMS (Danish Sports Medicine Society) guidelines and the DRS (Danish Rheumatological Society) guidelines.
Consideration when participating in the experiment:
Patients in the trial will be offered more thorough instruction in load reduction and prolonged physiotherapeutic supervised training. In addition, there will be a more thorough follow-up of the patients than can usually be offered.
Half of the patients are treated with steroid injections. If these prove to be of value, there is a clear benefit for patients here. The disadvantage of both groups receiving injection is the discomfort of injection, as well as the risk of serious side effects, which, however, as stated in the chapter "risk assessment", is small.

12.1 Risks

Risks divided for each of the groups.
Group 1: Exercise + local anesthetic injection.
There are no risks associated with the training. With very pronounced tenderness, there may be a habituation period of a few days, where a slight tenderness may be experienced.

Heavy strength training has in recent years proved to be a good tool for chronic tendon problems such as ligamentum patellae tendinopathy and Achilles tendinopathy. It is expected that a soreness is experienced during the performance of the exercise, but that this soreness decreases rapidly after the end of training.
Injection.
There will be a risk of discomfort and pain after the injection. The pain usually disappear after 1-2 days, but there may be soreness for a few weeks after the injection. It may be necessary for a short time to take mild painkillers (eg Paracetamol) for this.
Every time an injection is given, there is a risk of infection. The risk is estimated to be less than 1: 25,000 injections and can be treated with antibiotics if necessary.
Furthermore, there will be an extremely limited risk of the needle damaging a small nerve, which can cause - usually transient - sensory disturbance in the heel region.

Group 2: Exercise + steroid injection
Training: see above.
Steroid injection: Glucocorticosteroid injection may result in some patients (10%) experiencing exhilaration and / or severe redness of the face, as if you have had too much sun. It is harmless and disappears after a few days. In rare cases, some of the glucocorticosteroid may seep into the skin and cause the fat cells to "shrink". This can result in a bright and sunken area. This has only cosmetic significance, gives no inconvenience and will often disappear on its own. However, the change can last for several years. In the rare cases where adipose tissue atrophy occurs, one will not be able to keep it blind.

13. Safety assessment

Before injection, check for the contraindications, wounds in the area, infection in the area as well as insulin-requiring diabetes. Also checked for relative contraindication which is AK treatment.
All adverse reactions to the injections are recorded by subsequent medical examination.

17. Literature

Alfredson H, Pietilä T, Jonsson P, Lorentzon R. Heavy-Load Eccentric Calf Muscle Training For The Treatment Of Chronic Achilles Tendinosis. Am J Sports Med. 1998; 26: 360-6

Beyer R et al. Equal effect of eccentric exercises and heavy slow resistance in Achilles tendinopathy. Poster Danish Sportsmedicine congress. 2015

Fredberg U, Bolvig L, Pfeiffer-Jensen M, Clemmensen D, Jakobsen BW, Stengaard-Pedersen K. Ultrasonography as a tool for diagnosis, guidance of local steroid injection and, together with pressure algometry, monitoring of the athletes with chronic jumper's knee and Achilles tendinitis: a randomized, double-blind, placebo-controlled study. Scand J Rheumatol. 2004; 33: 94-101

Johannsen FE, Gam AN. Achillodynia is not just a sports injury. Ugeskr Laeger 2010; 172: 3325-9.

Khan KM, Cook JL, Kannus P, Maffulli N, Bondesteam S. Time to abandon the “tendonitis” myth (editorial). BMJ 2002; 324: 626-7.

Kujala UM, Sarna S, Kaprio J Cumulative incidence of Achilles tendon rupture and tendinopathy in male former elite athletes. Clin J Sport Med. 2005 May; 15 (3): 133-5.

Kvist M. Achilles tendon injuries in athletes. Ann Chir Gynaecol. 1991; 80 (2): 188-201

Lysholm J, Wiklander J. Injuries in runners. Am J Sports Med. 1987 Mar-Apr; 15 (2): 168-71.

Mafi N, Lorentzon R, Alfredson H. Superior short-term results with eccentric calf muscle training compared to concentric training in a randomized prospective multicenter study on patients with chronic Achilles tendinosis. Knee Surg, Sports Traumatol, Arthrosc. 2001; 9: 42-7

Maffulli N, Walley G, Sayana MK, Longo U G, Denaro V. Eccentric calf muscle training in athletic patients with Achilles tendinopathy. Disability and Rehabilitation. 2008; 30 (20-22): 1677-84

Maffulli N, Longo U G, Loppini M, Denaro V. Current treatment options for tendinopathy. Expert Opin.Pharmacother. 2010; 111 (13): 2177-86

Malliaras P, Barton CJ, Reeves ND, Langberg H. Achilles and Patellar Tendinopathy Loading Programs. Asystematic Review Comparing Clinical Outcomes and Identifying Potential Mechanisms for Effectiveness. Sports Med. 2013; 43: 267-86

Nørregård J, Larsen CC, Bieler T, Langberg H. Eccentric exercise in treatment of Achilles tendinopathy. Scand J Med Sci Sports. 2007; 17: 133-8.

Ohberg L, Alfredson H. Effects on neovascularization behind the good results with eccentric training in chronic mid-portion Achilles tendinosis? Knee Surg Sports Traumatol Arthrosc. 2004 Sep; 12 (5): 465-70.

Porter D, Barrill E, Oneacre K, May BD. The effects of duration and frequency of Achilles tendon stretching on dorsiflexion and outcome in painful heel syndrome: a randomized, blinded, control study. Foot Ankle Int. 2002 Jul; 23 (7): 619-24.

Ram R, Meeuwisse W, Patel C, Wiseman DA, Wiley JP. The limited effectiveness of a home-based eccentric training for the treatment of Achilles tendinopathy. Clin Invest Med. 2013 Aug 1; 36 (4): E197-206.

Silbernagel KG, Thomeé R, Thomeé P, Karlsson J. Eccentric overload training for patients with chronic Achilles tendon pain - a randomized controlled study with reliability testing of the evaluation methods. Scand J Med Sci Sports. 2001; 11: 197-206

Wetke E, Johannsen F, Langberg H. Achilles tendinopathy: A prospective study on the effect of active rehabilitation and steroid injection in a clinical setting. Scand J Med Sci Sport 2014; doi: 10.1111 / sms.12326.

STATISTICAL ANALYSIS PLAN

Heavy weight training as a treatment for Achilles tendinopathy supplemented with injection of glucocorticosteroid or local anesthetics.

Trial Registration

Clinicaltrials.gov Trial registration identifier: NCT02580630
Ethical Committee of the Capital Region: H-15006579

Protocol Version and Date

This document has been written based on information contained in the study protocol version 3, approved by the Ethical Committee.

Statistical Analysis Plan Version and Date

Version 2
4th May, 2021

Statistical Analysis Plan Authors

Finn Johannsen
Michael Rathleff
Jens Lykkegaard Olesen
Peter Magnusson

1 SIGNATURES

Approved by Finn Johannsen

Finn Johannsen, MD.
Bispebjerg Hospital

[signature]
Signature

4/5-2021
Date

Approved by Michael Rathleff

Michael Rathleff, Professor, PT, MSc,PhD,
Center for General Practice,
Aalborg University

Michael S. Rathleff
Signature

21/1/2022
Date

Approved by Jens Lykkegaard Olesen

Jens Lykkegaard Olesen, Professor, MD, PhD.
Center for General Practice,
Aalborg University

[signature]
Signature

21/1/22
Date

Approved by Peter Magnusson

Peter Magnusson, Professor, PT, PhD, DMSc
Institute of Sports Medicine
University of Copenhagen

S. Peter Magnusson
Signature

21/1/22
Date

2 STUDY METHODS

2.1 Trial Design

Triple blinded Randomized Controlled Trial.

2.2 Sample Size and Power

There is no established minimal clinically important change (MCID) in the VISA-A score for the mid portion Achilles tendinopathy (Iversen JV et al, 2012) however MCID for insertion Achilles tendinopathy is found to be 6 points (McCormack J, 2015). The study will be powered to detect a between-group difference in the VISA-A score of 10 points after the primary endpoint at 6 months. Using the mean VISA-A score from Beyer et al (2015) at 3 and 12 months, a type I error rate of 5% and a type II error rate of 20% (80% power), we will need 47 patients in each study arm for a 2-sample t-test of a normal mean difference with a 2-sided significance level of 0.05, assuming a common standard deviation of 16 points.

To allow for a 10% potential dropout and a larger standard deviation than previous trial, we will increase the sample-size to 50 patients per group and include a total of 100 patients. The primary analysis will be blinded and follow the intention-to-treat principle with baseline values as covariates. A secondary per-protocol analysis may be performed. No interim analyses will be made.

2.3 Blinding

Randomization is performed by an independent office employee (Trine Stefanski) using a computer generated randomization schedule (MINIMPY, Maghaei 2010) using permuted block sizes (two to six). The employee is blinded to the block sizes. The allocation of each patient is stored in a computer drive with exclusive access only for the responsible office employee (Trine Stefanski).

All patients are given consecutive research numbers.

Patients are blinded to treatment: The syringe are blinded with tape only showing the patients research number. The content is visually identical and with similar viscosity by mixing intralipid to the placebo injection.

Doctors performing the injections are blinded (FJ, JLO, TØ): The syringe are blinded with tape only showing the patients research number. The content is visually identical and with similar viscosity by mixing intralipid to the placebo injection.

Data entry investigator are blinded. An independent investigator (ASJ) not involved in the patient treatment and without knowledge of treatment group allocation performes the data entry from Clinical Research Files.

Statistician is blinded (PSM): After data entry, the patients are divided in two groups: group 1 and group 2 by help from independent office employee. The primary investigator will group the patients in the two groups still blinded to the treatment group. All statistics are performed until agreement of the results. Thereafter the blinding is broken.

3 OUTCOMES

3.1 Primary outcome

The primary outcome measure is the change in the total score of the Victorian Institute of Sport assessment Achilles (VISA-A) questionnaire at 6 months compared to baseline, with additional secondary endpoints in VISA-A at other timepoints (1,2,3,12,24 months).

3.2 Key Secondary outcomes

The following outcomes are assessed as key secondary outcomes:

Morning pain on a 100 mm Visual Analogue Scale (VAS)

Pain during activity on a 100 mm Visual Analogue Scale (VAS)

Ultrasonography measurement of the anterior-posterior thickness of the Achilles tendon

Ultrasonography measurement of the colour doppler in the Achilles tendon graded 0-3.

Patient rated return to sport compared to sports activity before the injury

Patient self reported Global rating of Change on a Lickert scale from -5 to +5, where 0 is unchanged from baseline and +5 is completely cured

3.3 Adverse events

The adverse effects committee is chaired by JLO. All adverse events, defined as any negative or unwanted reactions to the interventions will be recorded. A patient diary will be given to patients at baseline where patients are asked for any adverse events during the first three months. This will capture additional ill effects not reported at the time of their occurrence.

3.4 Data collection

Three outcome assessors will perform all enrollment, baseline and follow-up assessments. Before starting the data collection, the assessors will train together and decide on a consensus standard of inclusion criteria and all interpretation of all outcome variables. This will ensure a uniform inclusion and follow-up of participants.

4 STATISTICAL ANALYSES

4.1 General

We originally planned to do 2-way ANOVA, but due to many dropouts and therefore missing values, we have decided in this version 2 to use mixed effect model analysis, rather than by repeated measures ANOVA (which can't handle missing values).

4.2 Statistical Analysis Plan

Statistical analysis will be undertaken using PRISM version 9.3.1. All analyses will be conducted on an intention-to-treat principle using all randomised participants in the groups they were originally randomised to. Missing data will be multiply imputed, due to an expected strong time trend. A blinded statistician will perform the data analysis.

Demographic and anthropometric characteristics (gender, age, mass, height, body mass index, sporting activities and pain duration) will be determined at the baseline visit for each treatment group.

Statistical analyses will be conducted on 1, 2, 3, 6, 12 and 24-month outcome measures. However, the primary end-point will be change in the total score of the VISA-A questionnaire at 6 months. Between-group comparisons of treatment effect for all primary and secondary outcomes, will be performed with a mixed-effects model, with patient as a random effect and time of assessment (baseline, 1, 2, 3, 6, 12 and 24-month), study group (Intervention: HSR and corticosteroid injection or control: HSR and placebo injection), and baseline values of the outcome as fixed effects. Interaction between time of assessment and study group will also be included in the model. Crude analyses and analyses adjusted for time of assessment,

baseline values of the outcome, and the interaction between time of assessment and study group are performed. To assess for superiority, mean between-group differences in changes from baseline and two-sided 95% confidence intervals will be calculated.

The ordinal scaled data of patients overall assessment (Lickert score -5 to +5) will be analyzed with the same mixed effect model, if the data is found normally distributed.

The ordinal scaled data of grading the flow within the tendon into 4 categories will be calculated using non parametric statistics: Mann-Whitney U-tests.

A two-sided P value of less than 0.05 will be considered to indicate statistical significance.

4.3 Interim analysis and early stopping rules

Anecdotally, an increased risk of tendon rupture have been reported. To account for this, we have introduced an early stopping rule if the rate of Achilles tendon ruptures exceed 2 ruptures.

4.4 Timing of analyses

When this statistical analysis plan was signed, recruitment to the main trial was completed, but analysis had not been initiated. Statistical analyses are expected to be completed after 3-6 months.

	Enrolment	Allocation						
**TIMEPOINT****	april 2016 – end 2018	april 2016 – end 2018	*Month 1*	*Month 2*	*Month 3*	*Month 6*	*Month 12* *April 2017* *end 2019*	Month 24 April 2018 end 2020
**ENROLMENT:**								
**Eligibility screen**	X	X						
**Informed consent**		X						
**Allocation**		X						
**INTERVENTIONS:**								
Intervention group			◆———	———	———◆			
Control group			◆———	———	———◆			
**ASSESSMENTS:**								
Diagnosis		X						
VISA-A		X	X	X	X	X	X	X
Morning pain [VAS]		X	X	X	X	X	X	X
Pain after activity [VAS]		X	X	X	X	X	X	X
Return to usual sports participation						X	X	X
Satisfaction with result of treatment			X	X	X	X	X	X
Patient diary for reporting compliance and adverse events			◆———	———	———◆			
Ultrasound examinations		X	X	X	X	X	X	X
Demographics		X						

Table 1: SPIRIT figure. Schedule of enrolment, interventions and assessments

5 DEVIATIONS FROM THE PROTOCOL

The following details in this SAP represents deviations from protocol version 3

Header in protocol	Change	Reason
Repetition of injection	Changed from Based on tissue structure In Ultrasound scanning To "Injection is given every months until the tendon pain is markedly diminished (max 3 injections)"	More clinical relevant. And we wanted to limit the number of injections. This is included in clinicaltrials.gov before study start, but corrected in accepted prtocol
9.2 Secondary outcomes	Return to sport compared to sports activity before injury is cancelled	Many have long lasting injury and difficulty in recalling former activity.
24 months evaluation	Extended	Due to Corona pandemic
Statistical method	Due to many dropouts and the wish of intention to treat, we will change the analysis to mixed method analysis instead of 2 way ANOVA.	4th May 2021. With still blinded data
