# Supplementary material for: Effect of Ultrasonography-Guided Corticosteroid Injection vs Placebo Added to Exercise Therapy for Achilles Tendinopathy: A Randomized Clinical Trial
Source: JAMA Netw Open. 2022 Jul 11;5(7):e2219661. doi: 10.1001/jamanetworkopen.2022.19661 (PMC9274322; doi:10.1001/jamanetworkopen.2022.19661)
Supplement: Supplement 2. — eFigure 1. Secondary Outcome: Improvement in VAS-Morning Pain From Baseline to 1, 2, 3, 6, 12, and 24 Months eFigure 2. Secondary Outcome: Improvement in VAS-Activity Pain From Baseline to 1, 2, 3, 6, 12, and 24 Months eFigure 3. Secondary Outcome: Likert Score at All Time Points eTable 1. Likert Score (-5 to +5) Divided in Groups at All Time Points eTable 2. Likert Score: Number of Patients Much Improved or Cured at All Time Points eTable 3. Likert Score: Mean and 95% CI at All Time Points [file jamanetwopen-e2219661-s002.pdf]

Supplemental Online Content

Johannsen F, Olesen JL, Øhlenschläger TF, et al. Effect of ultrasonography-guided corticosteroid injection vs placebo added to exercise therapy for Achilles tendinopathy: a randomized clinical trial. *JAMA Netw Open*. 2022;5(7):e2219661. doi:10.1001/jamanetworkopen.2022.19661

eFigure 1. Secondary Outcome: Improvement in VAS-Morning Pain From Baseline to 1, 2, 3, 6, 12, and 24 Months

eFigure 2. Secondary Outcome: Improvement in VAS-Activity Pain From Baseline to 1, 2, 3, 6, 12, and 24 Months

eFigure 3. Secondary Outcome: Likert Score at All Time Points

eTable 1. Likert Score (-5 to +5) Divided in Groups at All Time Points

eTable 2. Likert Score: Number of Patients Much Improved or Cured at All Time Points

eTable 3. Likert Score: Mean and 95% CI at All Time Points

This supplemental material has been provided by the authors to give readers additional information about their work.

© 2022 Johannsen F et al. *JAMA Network Open.*

eFigure 1. The Secondary Outcome: Improvement in VAS-Morning Pain From Baseline to 1, 2, 3, 6, 12, and 24 Months. The I bars indicate the 95% CI. Stars indicate significant differences.

VAS morning pain

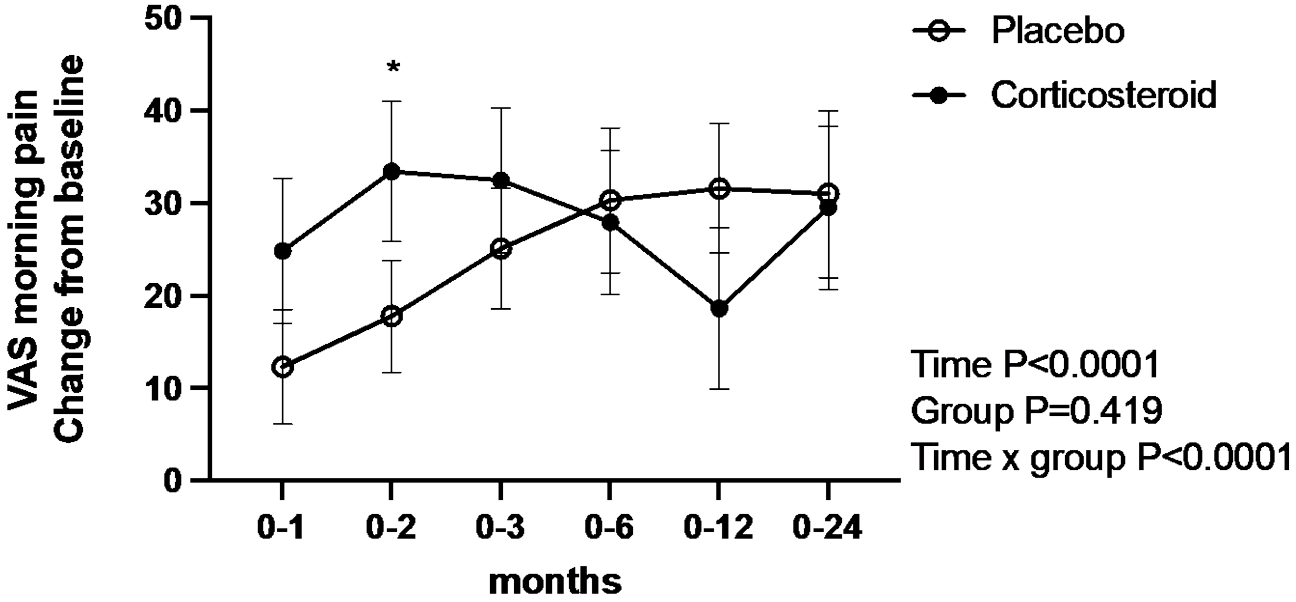

© 2022 Johannsen F et al. *JAMA Network Open.*

eFigure 2. Secondary Outcome: Improvement in VAS-Activity Pain From Baseline to 1, 2, 3, 6, 12, and 24 Months. The I bars indicate the 95% CI. Stars indicate significant differences.

VAS activity pain

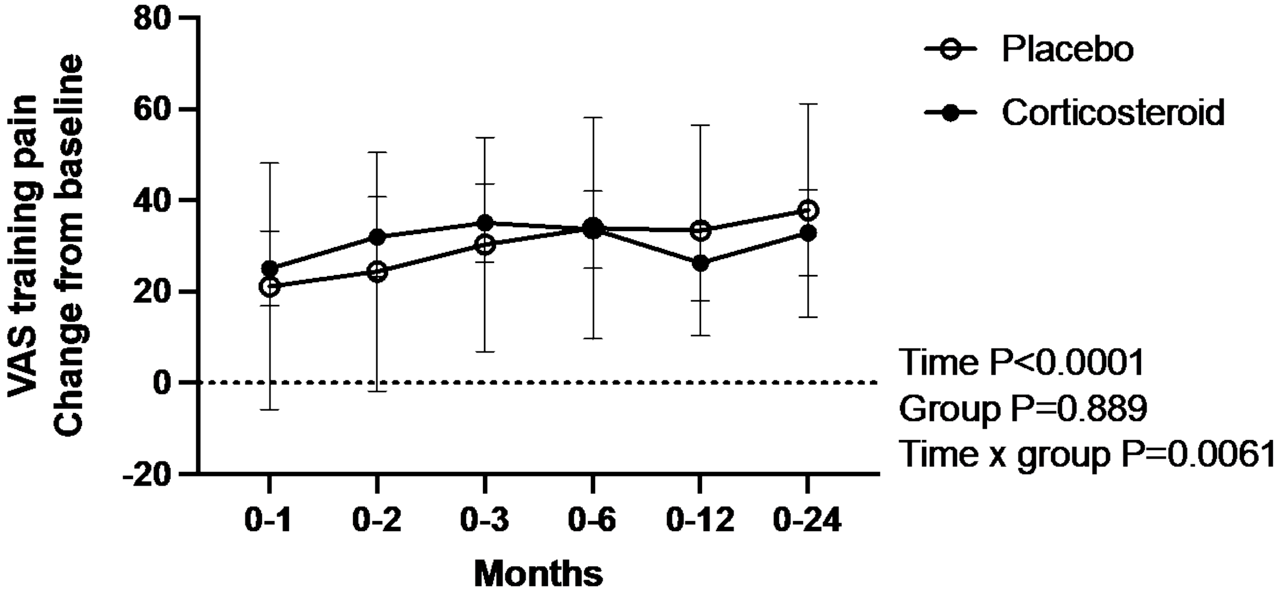

© 2022 Johannsen F et al. *JAMA Network Open.*

eFigure 3. Secondary Outcome: Likert Score at All Time Points. 0 unchanged from entry, 1-2 light improvement, 3-4 much improvement, 5 cured. Negative values are worsening. The I bars indicate the 95% CI. Stars indicate significant differences.

Likert score

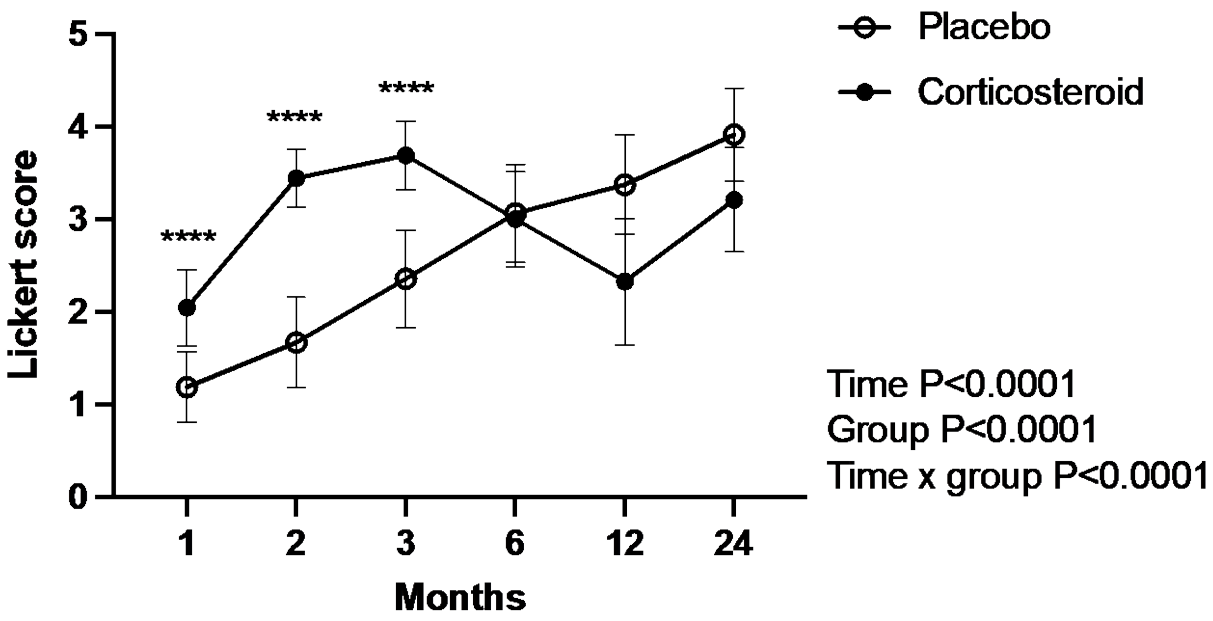

© 2022 Johannsen F et al. *JAMA Network Open.*

eTable 1. Likert Score (-5 to +5) Divided in Groups at All Time Points

0 unchanged from entry, 1-2 light improvement, 3-4 much improvement, 5 cured. Negative values are worsening

	Corticosteroid				Placebo			
Likert score	=< 0 Unchanged/ worsend	1-2 Slight improvement	3-4 Much improvement	5 cured	=< 0 Unchanged/ worsend	1-2 Slight improvement	3-4 Much improvement	5 cured
1 month	8/45	18	18	1	15/48	26	7	0
2 months	1/45	6	33	5	15/48	15	17	1
3 months	2/45	3	29	11	10/48	11	23	4
6 months	3/42	8	25	6	6/48	9	22	11
12 months	9/37	6	18	4	5/42	6	17	14
24 months	2/30	5	15	8	2/34	2	16	15

eTable 2. Likert Score: Number of Patients Much Improved or Cured at All Time Points

	Corticosteroid cured/ much improved	Placebo cured/ much improved
1 month	19/45 (42%)	7/48 (15%)
2 months	38/45 (84%)	18/48 (38%)
3 months	40/45 (89%)	27/48 (56%)
6 months	31/42 (74%)	33/48 (69%)
12 months	22/37 (59%)	31/42 (74%)
24 months	23/30 (77%)	31/34 (91%)

eTable 3. Likert Score: Mean and 95% CI at All Time Points

month	Placebo mean	95% CI Upper limit	Lower limit	Corticosteroid mean	95% CI Upper limit	lower limit
1	1,184	1,567	0,801	2,043	2,454	1,633
2	1,667	2,158	1,175	3,444	3,755	3,134
3	2,354	2,878	1,83	3,689	4,056	3,322
6	3,063	3,593	2,532	3	3,516	2,484
12	3,372	3,909	2,835	2,325	3,006	1,644
24	3,914	4,417	3,412	3,212	3,778	2,646

© 2022 Johannsen F et al. *JAMA Network Open.*
