# Supplementary material for: Effect of Ultrasonography-Guided Corticosteroid Injection vs Placebo Added to Exercise Therapy for Achilles Tendinopathy: A Randomized Clinical Trial
Source: JAMA Netw Open. 2022 Jul 11;5(7):e2219661. doi: 10.1001/jamanetworkopen.2022.19661 (PMC9274322; doi:10.1001/jamanetworkopen.2022.19661)
Supplement: Supplement 3. — Data Sharing Statement [file jamanetwopen-e2219661-s003.pdf]

Data Sharing Statement

Johannsen F, Olesen JL, Øhlenschläger TF, et al. Effect of ultrasonography-guided corticosteroid injection vs placebo added to exercise therapy for Achilles tendinopathy: a randomized clinical trial. *JAMA Netw Open*. 2022;5(7):e2219661. doi:10.1001/jamanetworkopen.2022.19661

Data
Data available: No

Additional Information
Explanation for why data not available: due to European GDPR law
